# The association of dietary pattern and breast cancer in Jiangsu, China: A population-based case-control study

**DOI:** 10.1371/journal.pone.0184453

**Published:** 2017-09-12

**Authors:** Shurong Lu, Yun Qian, Xingyu Huang, Hao Yu, Jie Yang, Renqiang Han, Jian Su, Wencong Du, Jinyi Zhou, Meihua Dong, Xiaojin Yu, Fränzel J. B. van Duijnhoven, Ellen Kampman, Ming Wu

**Affiliations:** 1 Department of Chronic Disease Control, Jiangsu Provincial Center for Disease Control and Prevention, Nanjing, Jiangsu, China; 2 Department of Chronic Disease Control, Wuxi Center for Disease Control and Prevention, Wuxi, Jiangsu, China; 3 School of Public Health, Southeast University, Nanjing, Jiangsu, China; 4 Division of Human Nutrition, Wageningen University, Wageningen, The Netherlands; Institute for Nutritional Sciences, CHINA

## Abstract

This study aims to examine the association of breast cancer with dietary patterns among Chinese women. A population-based case-control study was conducted in Jiangsu, China. Newly diagnosed primary breast cancer patients were recruited as cases (n = 818). Controls (n = 935), selected from the general population, were frequency matched to cases. A validated food frequency questionnaire was used to assess dietary intake. Dietary patterns were identified by factor analysis and multivariable odds ratios (OR) and 95% confidence intervals (CI) were estimated. Four dietary patterns were identified: salty, vegetarian, sweet and traditional Chinese. The traditional Chinese pattern was found to be robustly associated with a lower risk of breast cancer among both pre- and post-menopausal women (4^th^ *vs*. 1^st^ quartile: OR for pre- and post-menopausal women was 0.47 and 0.68, respectively). Women with high factor scores of the sweet pattern also showed a decreased risk of breast cancer (4^th^ *vs*. 1^st^ quartile: OR for pre- and post-menopausal women was 0.47 and 0.68, respectively). No marked association was observed between a vegetarian pattern or a salty pattern and breast cancer. These findings indicate that dietary patterns of the traditional Chinese and the sweet may favorably associate with the risk of breast cancer among Chinese women.

## Introduction

Breast cancer is the most common cancer in women in high-income countries and its incidence and mortality have been rising in low- to middle-income countries [1, 2]. In China, on average, the incidence rate of breast cancer is 5.3 times lower than that in North America (age-standardized incidence rate 18.7 *vs*. 99.4 per 100,000). However, there has been a remarkable increase in recent years [3, 4], and breast cancer has now been listed as one of the priorities for female cancer prevention in China [5, 6].The increase in incidence and mortality observed in low- to middle-income countries is generally thought to be related to a variety of factors such as aging, lifestyle changes, and migration to urban communities [7]. The rising incidence of breast cancer in parallel with economic development in China indicates that diet, which has changed gradually over the past three decades [8], might contribute to the risk of breast cancer.

So far, epidemiologic studies addressing the role of dietary patterns in breast cancer development were mostly performed in Western countries such as North America [9–11] and Europe [12, 13]. Dietary patterns are likely to vary among different populations due to geographic differences, socioeconomic status, and culture in food habits, preferences, and availability [14]. Several studies performed in Asian populations [15–19] suggested that certain dietary patterns, for example, a western pattern or a diet characterized by vegetables, fruit, and soy, could influence the risk of breast cancer among Asian women. More specifically, a limited number of studies performed in different areas of China [15, 18, 19] indicated that dietary patterns were associated with breast cancer, but no consistency in results was achieved. For example, Cui *et al* [15] reported that a vegetable-soy pattern was not associated with breast cancer risk, whereas Zhang *et al* [18] found that high consumption of a vegetable/fruit/soy pattern could decrease the risk. As one of the most important dietary patterns in the world, the traditional Chinese diet is characterized by balanced intake of vegetables and meats, more consumption of starchy foods, a quantity intake of soy and aquatics, as well as its cooking methods of stir-frying or simmering. Yet this typical pattern has not been analyzed in either of the previous studies. Therefore, we conducted this population-based case—control study to identify the major dietary patterns among Chinese women and to examine the relationship between these patterns and breast cancer risk.

## Materials and methods

### Study area, participants and data collection

A population-based case-control study on breast cancer was conducted in Wuxi City of Jiangsu Province during 2013–2014. Jiangsu is a province in the southeast of China with a population of 80 million, while Wuxi is in the south of Jiangsu and is located along the Yangtze River between Shanghai and Nanjing, with a population of 6.5 million.

All study subjects were restricted to local female inhabitants who had lived in Wuxi for at least 5 years. Using data from the local population-based cancer registry, patients who were (1) being newly diagnosed with primary breast cancer within 12-month; (2) being diagnosed based on pathological and/or iconographic evidence; and (3) being identified by the International Classification of Diseases, tenth revision (ICD—10, code C50), were recruited as cases. Second primary and recurrent breast cancer, as well as individuals with a history of any cancer, were excluded. Eligible controls were randomly selected from the general population of the same area as cases, using the data of the city demographic database. Controls and cases were frequency matched by age (± 5-year). During the study period, 1,410 new breast cancer cases were identified and 864 (61.3%) of them were recruited, with 818 patients with complete information were included in this analysis. Meanwhile, a number of 1,072 controls were invited and 935 joined the study, with a response rate of 87.2%.

All eligible persons were invited to Wuxi Maternal and Child Health Hospital, which is the largest women and children healthcare hospital in Wuxi City. Using standard protocols and structured questionnaires, epidemiologic data was collected by trained investigators through face-to-face interviews. The questionnaire included detailed information on known or potential risk or protective factors for breast cancer, including demographic information, socio-economic status, dietary habits, reproductive history, disease history, *etc*. Following the interview, anthropometric measurements on height and weight [used to calculate body mass index (BMI, kg/m^2^) as weight (kg) divided by squared height (meters)], waist and hip circumference [used to calculate waist-hip ratio (WHR) as waist circumference (cm) divided by hip circumference (cm)] were conducted.

This study was approved by the Ethical Committee of Jiangsu Provincial Center for Disease Control and Prevention. All participants were fully informed and gave written consent.

### Dietary pattern assessment

An interviewer-administrated food frequency questionnaire was used to collect information of individual food consumption in the previous 12 months. The validity and the reliability of this questionnaire in terms of food consumption have been documented elsewhere [20]. This questionnaire included 23 groups of foods and beverages which covered majority of common foods consumed in China. Three aspects of each individual item were listed in the questionnaire, including whether the item was consumed or not, if yes, what the usual frequency of consumption (number of times per day/week/month/year) was and what the estimated average amount of food eaten each time was, using a local unit liang for weight (1 liang = 50 g) or cup for capacity (1 cup = 250 ml). A common unit or portion size for each food was specified by reference to food models. Intakes of foods and beverages were converted into g/d or ml/d by frequency timing average amount.

Exploratory factor analysis was used to identify dietary patterns using principal component analysis approach [21]. The factors were rotated by an orthogonal transformation to improve interpretability and minimize the correlation between factors. The number of factors retained from each food classification method was determined by multiple criteria including factor interpretability, the diagram of eigenvalues, scree plot, and the amount of variance explained by each factor [21]. Factor scores are equivalent to a simple correlation between the food items and the factor. Higher scores indicate that the food item shares more variance with that factor. The sign of the scores determines the direction of the relationship of each food to the factor [14, 21]. Labeling of the derived factors was primarily descriptive and based on our interpretation as well as of the input variable with the highest factor loading.

### Statistical analysis

Factor scores of dietary patterns were divided into quartiles to allow a robust estimation of the associations which were not driven by extreme values. The chi-squared test was used to compare the difference between categorical variables. In our study, multivariate logistic regression was applied to assess the odds ratio (OR) and their 95% confidence intervals (CI) of breast cancer risk by quartiles of each dietary pattern factor scores. Age (continuous), education level (Illiterate, low, medium and high), household income 5 years ago (continuous) and residence (urban or rural) were selected as potential confounders based on the comparison of baseline characteristics between case and control subjects, as well as on the results of literature review. As menopausal status is one of the most important known confounders in breast cancer related analyses [22], all analyses were stratified by menopausal status (pre- and post-menopause) in this study. To test the possible effect of obesity (overall, measured with BMI; and central, with WHR), we further proceeded stratified analysis by BMI (< 28 kg/m^2^ and > = 28 kg/m^2^) and WHR (< 0.8 and > = 0.8) in the logistic regression model. Reproductive related variables, including age at menarche (continuous), age at first live birth (continuous) and abortion (yes or no) were additionally introduced into the model in order to see their influence on the association.

SPSS (statistics standard, SPSS Inc, Chicago, Illinois, USA) was used for data analyses. All *P* values were two sided, and statistical significance was determined at the *P* < 0.05 level.

## Results

Descriptive characteristics of the population by case-control status are shown in Table 1. Compared with controls, cases were more likely to have a lower household income (19.1 thousand *vs*. 24.8 thousand, *P* < 0.001) but higher proportion of illiterate to low education (*P* < 0.001). The case group was higher in both average BMI and WHR than the control group (both *P* < 0.001). Though there was no statistically significant difference either in age of menarche or age of first live birth between cases and controls, the case group reported more frequently to be post-menopausal (*P* < 0.001), but they reported a lower percentage of abortion experience (13.42% *vs*. 17.93%, *P* = 0.018).

**Table 1:** Descriptive characteristics of the population by case/control status.

			Cases (n = 818)	Controls (n = 935)	*P*
General information	Current age	years, mean(sd)	54.8(11.1)	54.3(11.3)	*0*.*337*
	Household income	Ұ1,000, mean(sd)	19.1(22.0)	24.8(22.6)	*<0*.*001*
	Residence (n, %)	Urban	446(54.7)	511(55.2)	*0*.*847*
		Rural	369(45.3)	415(44.8)	
	Education (n, %)	Illiterate	53(6.5)	47(5.0)	*<0*.*001*
		Low	203(24.9)	181(19.4)	
		Medium	496(60.7)	566(60.7)	
		High	65(8.0)	139(14.9)	
Weight indices	Body mass index	kg/m2, mean(sd)	24.9(3.5)	24.1(3.2)	*<0*.*001*
	Waist hip ratio	mean(sd)	0.9(0.1)	0.9(0.1)	*<0*.*001*
Fertility indices	Age at menarche	years, mean(sd)	15.6(1.9)	15.6(1.9)	*0*.*998*
	Age of first live birth	years, mean(sd)	24.6(2.7)	24.6(2.5)	*0*.*874*
	Menopausal (n, %)	Yes	386(41.3)	268(32.8)	*<0*.*001*
		No	549(58.7)	550(67.2)	
	> = One abortion (n, %)	Yes	102(13.4)	125(17.9)	*0*.*018*
		No	658(86.6)	572(82.1)	

In principal components analysis, four dietary patterns were identified. Factor scores of the four patterns and the name assigned to each pattern are presented in Table 2. The salty pattern (factor 1) loaded heavily on high-in-salt food, including salt, soy sauce, monosodium glutamate *etc*. The vegetarian pattern (factor 2) largely consisted of more vegetarian food, such as soy, fruits and coarse cereals, but contained less meat and fried food. The sweet pattern (factor 3) was characterized by various kinds of sweet foods or beverages, such as soft drinks, sugar strengthened beverages and cakes. The traditional Chinese pattern (factor 4) mainly includes meat, vegetable/fruit, whole cereal (food made from rice, wheat flour and corn, *etc*.) and eggs. The four factors explained 32.9% of the variance in food intake, with patterns of salty, vegetarian, sweet and traditional Chinese patterns accounting for 11.72%, 11.32%, 7.53% and 5.76%, respectively.

**Table 2:** Dietary patterns, groups of food/ beverage and factor scores^a^ (N = 1753).

Salty (Factor 1)		Vegetarian (Factor 2)		Sweet (Factor 3)		Traditional Chinese (Factor 4)	
Groups of food/ beverage	Factor scores^b^	Groups of food/ beverage	Factor scores	Groups of food/ beverage	Factor scores	Groups of food/ beverage	Factor scores
Salt	0.76	Beans	0.87	Soft drinks	0.62	Meat	0.59
Oil	0.74	Nuts	0.87	SSB	0.51	Eggs	0.51
Monosodium glutamate	0.70	Fruits	0.76	Fried food	0.44	Rice/Flour	0.51
Soy sauce	0.69	Vegetables	0.52	Cakes	0.44	Aquatics	0.49
Sugar	0.69	Aquatics	0.27	Milk	0.41	Vegetables	0.40
Soft drinks	0.07	Milk	0.19	Coffee	0.38	Poultry	0.32
Pickles	0.07	Cereals	0.13	Fresh juice	0.37	Pickles	0.17
Cereals	0.06	Rice/Flour	0.08	Eggs	0.29	Fruits	0.15
Rice/Flour	0.05	Fresh juice	0.07	Poultry	0.23	Milk	0.11
Fried food	0.04	Meat	0.07	Cereals	0.19	Fried food	0.10
SSB^c^	0.02	Cakes	0.02	Beans	0.10	Sugar	0.07
Aquatics	-0.03	Soft drinks	-0.02	Nuts	0.09	Oil	0.03
Coffee	-0.04	Poultry	-0.02	Fruits	0.08	SSB	0.03
Eggs	-0.05	SSB	-0.03	Soy sauce	0.07	Salt	0.03
Fruits	-0.06	Eggs	-0.06	Aquatics	0.05	Cakes	0.03
Fresh juice	-0.07	Pickles	-0.07	Meat	0.05	Monosodium glutamate	0.03
Milk	-0.07	Fried food	-0.07	Salt	-0.03	Cereals	-0.02
				Oil	-0.05	Beans	-0.05
				Vegetables	-0.16	Soft drinks	-0.06
				Rice/Flour	-0.27	Nuts	-0.10

^a^ Only groups of food or beverage with absolute values > 0.02 were listed for simplicity.

^b^ Factor scores are equivalent to a simple correlation between the food items and the factor. Higher scores indicate that the food item shares more variance with that factor. The sign of the scores determines the direction of the relationship of each food item to the factor.

^c^ SSB: Sugar strengthened beverage.

Compared with the lowest quartile (Q1) of the traditional pattern, the highest quartile (Q4) had a lower mean age but higher household income, so for the sweet pattern. Rural residents had a higher percentage of salty and traditional patterns in Q4, whereas urban residents had a higher percentage in Q4 of both salty and traditional patterns. High education group accounted for higher proportion in Q4 of all patterns but the salty. In terms of intakes of variety foods, the Q4 group of salty pattern consumed the most oil, salt, pickles and soy sauce across different quartiles of dietary patterns (37 g/d of oil, 7 g/d of salt, 6 g/d of pickles and 7 g/d of soy sauce, respectively). Similarly, the Q4 group of vegetarian pattern group had the most vegetables and fruits (37 g/d) and sweet pattern had most soft drinks (18 ml/d), sugar strengthened beverages (24 ml/d) and cakes (7 g/d). The highest intake of whole grains (387 g/d), poultry (99 g/d), aquatics (21 g/d) and eggs (83 g/d) were found in the fourth quartile of the traditional Chinese pattern (Table 3).

**Table 3:** Sample characteristics for the lowest (Q1) and highest (Q4) quartiles of four dietary patterns (N = 1753).

		Salty^a^ (Factor 1)		Vegetarian^a^ (Factor 2)		Sweet^a^ (Factor 3)		Traditional Chinese^a^ (Factor 4)	
		Q1	Q4	Q1	Q4	Q1	Q4	Q1	Q4
Current age	years, mean(sd)	53.1(12.2)	56.1(11.1)	54.0(11.1)	53.8(10.6)	56.6(10.0)	52.3(11.8)*	56.0(11.5)	53.5(10.7)*
Household income	Ұ1,000, mean(sd)	22.1(25.0)	22.1(22.3)*	20.5(21.4)	23.9(21.7)*	18.8(19.4)	25.7(23.7) *	20.2(24.9)	23.5(19.4) *
Residence (n, %)	Urban	301(69.2)	194(44.7)	212(48.6)	259(59.5)	145(33.3)	313(72.5)	236(53.9)	219(50.3)
	Rural	134(30.8)	240(55.3)	224(51.4)	176(40.5)	291(66.7)	119(27.5)	202(46.1)	216(49.7)
Education (n, %)	Illiterate	23(5.3)	25(5.7)	30(6.9)	16(3.7)	42(9.6)	16(3.7)	34(7.8)	18(4.1)
	Low	78(17.8)	116(26.5)	110(25.2)	85(19.4)	133(30.4)	51(11.7)	107(24.5)	84(19.2)
	Medium	260(59.4)	275(62.8)	262(60.0)	264(60.3)	250(57.2)	276(63.2)	259(59.3)	269(61.4)
	High	77(17.6)	22(5.0)	35(8.0)	73(16.7)	12(2.7)	94(21.5)	37(8.5)	67(15.3)
Food intake (g/d, mean, sd)	Whole grains	275(131)	306(150)*	287(148)	310(251)*	392(288)	246(114)	219(106)	387(295)
	Vegetables & fruits	15(25)	22(43)*	9(18)	37(56)	9(18)	33(55)	21(46)	19(30)
	Animal meat	453(404)	457(285)*	254(144)	797(792)	571(587)	470(425)*	373(510)	644(648)
	Poultry	50(50)	55(66)*	53(60)	68(201)	52(196)	61(65)	25(28)	99(206)
	Aquatics	11(17)	12(18)*	14(24)	10(16)	6(10)	17(26)	4(7)	21(28)
	Eggs	47(149)	43(74)*	28(32)	76(169)	44(89)	57(148)	25(32)	83(172)
	Milk	30(33)	27(31)	34(38)	26(28)	14(21)	41(38)	10(14)	45(41)
	Cereals	85(128)	68(119)	38(83)	109(148)	10(54)	161(146)	56(101)	85(132)*
	Beans	15(22)	21(29)	9(13)	47(129)	14(23)	29(42)	25(124)	23(25)*
	Cakes	2(6)	3(9)*	5(12)	2(5)	0(1)	7(13)	1(6)	4(11)*
	Fried food	11(20)	12(32)*	9(29)	14(28)	2(5)	24(40)	9(23)	12(22)
	Soft drinks	5(30)	10(46)	10(44)	5(39)	<1	18(62)	9(47)	4(23)*
	SSB^#^	6(26)	9(30)*	12(62)	8(42)	1(4)	24(76)	6(32)	11(62)*
	Oil	1(4)	37(19)	19(19)	21(18)*	23(19)	18(17)	19(18)	21(19)*
	Salt	<1	9(5)	4(4)	5(4)	5(5)	4(4)	4(5)	4(4)*
	Pickles	4(8)	6(16)	7(16)	3(7)	5(15)	4(9)*	2(5)	8(17)
	Soy sauce	<1	7(6)	3(4)	4(5)	3(3)	4(4)	4(5)	3(4)*

^a^ The salty pattern loaded heavily on salt, soy sauce, monosodium glutamate; the vegetarian pattern largely consisted of soy, fruits and coarse cereals; the sweet pattern was characterized by more intake of soft drinks, sugar strengthened beverages and cakes; the traditional Chinese pattern includes mainly meat, vegetable/fruit, whole grains and eggs.

^#^ SSB: Sugar strengthened beverage.

* P < 0.05.

No association was observed between the salty pattern and the vegetarian pattern with breast cancer risk (Table 4). On the contrary, the risk significantly decreased in the highest quartile of the sweet pattern both in pre- and post-menopausal women (Q4 *vs*. Q1: OR among pre-menopausal women was 0.47, 95% CI: 0.28–0.79, *P* for trend = 0.017, OR among post-menopausal women was 0.68, 95% CI: 0.47–0.98, *P* for trend = 0.030). The traditional Chinese pattern (factor 4) was also found to be related to a lower risk of breast cancer and this association was not influenced by menopausal status. The ORs of breast cancer across quartiles of the traditional Chinese pattern among post-menopausal women were 0.64 (95% CI: 0.45–0.90) for Q2, 0.45 (95% CI: 0.32–0.63) for Q3 and 0.68 (95% CI: 0.48–0.97) for Q4, while those among pre-menopausal women were 0.53 (95% CI: 0.33–0.85), 0.53 (95% CI: 0.32–0.86) and 0.47 (95% CI: 0.29–0.76), respectively (Table 4).

**Table 4:** Odds ratio (OR) and 95% confidence interval (95% CI) of breast cancer across quartiles of four dietary patterns by menopausal status^a^.

		Pre-menopausal	Post-menopausal
Salty	Q1^b^	1.00(reference)	1.00(reference)
	Q2	1.22(0.77,1.93)	0.79(0.55,1.13)
	Q3	0.88(0.55,1.40)	0.66(0.46,1.00)
	Q4	1.41(0.87,2.29)	0.71(0.50,1.01)
	*P for trend*	0.392	0.052
Vegetarian	Q1	1.00(reference)	1.00(reference)
	Q2	1.09(0.68,1.75)	0.92(0.65,1.30)
	Q3	1.13(0.71,1.80)	0.97(0.68,1.38)
	Q4	1.57(0.98,2.49)	0.89(0.62,1.26)
	*P for trend*	0.067	0.589
Sweet	Q1	1.00(reference)	1.00(reference)
	Q2	0.55(0.33,0.90)	0.90(0.64,1.27)
	Q3	0.64(0.38,1.07)	0.80(0.56,1.13)
	Q4	0.47(0.28,0.79)	0.68(0.47,0.98)
	*P for trend*	0.017	0.030
Chinese-traditional	Q1	1.00(reference)	1.00(reference)
	Q2	0.53(0.33,0.85)	0.64(0.45,0.90)
	Q3	0.53(0.32,0.86)	0.45(0.32,0.63)
	Q4	0.47(0.29,0.76)	0.68(0.48,0.97)
	*P for trend*	0.004	0.006

^a^ Adjusted for age (continuous), education level (illiterate, Low, medium, high), household income (continuous) and residence (urban or rural).

^b^ Quartile 1 to quartile 4.

After stratification by BMI (< 28 kg/m^2^ and > = 28 kg/m^2^) and WHR (< 0.8 and > = 0.8), we found that the associations between traditional Chinese pattern, sweet pattern and breast cancer were still significant. By introducing reproductive related variables (age at menarche, age at first live birth and abortion) into the model, we observed that the highest quartile of sweet pattern, as well as the Q3 and Q4 quartiles of traditional Chinese pattern, were robustly related to a decreased risk of breast cancer, among both pre- and post-menopausal women (both *P* for trend < 0.05) (Data not shown).

## Discussion

In this population-based case—control study conducted in Jiangsu Province of China, we identified four major dietary patterns which were labeled as salty, vegetarian, sweet and traditional Chinese, respectively. The traditional Chinese pattern and the sweet pattern were found to be significantly associated with a reduced risk of breast cancer, and such associations were not influenced by menopausal status, weight status or reproductive history, whilst no marked associations were observed in the salty pattern or the vegetarian pattern. To our best knowledge, this is the first study that reports the association between traditional Chinese dietary pattern and breast cancer.

Contemporarily, there are three main dietary patterns throughout the world [23]: the Western pattern, represented by developed countries in North America and Europe, which is characterized by high intake of energy, fat and protein; the Eastern pattern, which is typical in Asian countries, consists of more vegetarian food but less animal food; and the Mediterranean dietary pattern, a typical diet in the Mediterranean region, which is rich in vegetables, fish, legumes and olive oil. Previous studies suggested that adhering to a Mediterranean diet could favorably decrease breast cancer risk [24, 25] and, conversely, the Western dietary pattern was related to elevated risk [2, 13, 26]. Since breast cancer rates are traditionally low among Asian women [1, 5, 6], not many studies have explored the effect of Eastern diet on breast cancer, except that some studies examined the association of certain foods which are commonly consumed in Eastern diet, such as vegetables, meat, fish or soy, with breast cancer [15–18]. A traditional Chinese diet is characterized by a balance in nutrients, containing diverse food groups such as whole grain (like rice and wheat flour), vegetables/fruits, meat and eggs to supply carbohydrates, proteins, fats and other indispensable nutrients [27].

Traditionally, sugar is not a major ingredient in a Chinese diet. However, sugar consumption has increased in the past decades among Chinese, especially among young people [8, 27]. Previous studies on single food item, *e*.*g*. sweet foods, and sugar, suggest that intakes of sweet items are associated with breast cancer, especially among young women [28]. This relation is hypothesized to be linked to high insulin exposure by large sugar intake and risk of breast carcinogenesis [29]. However, through dietary pattern analyses, no consistent results have been obtained on the association between sugar intake and breast cancer. For example, Nkondjock *et al* [10] reported that a chocolate-cereal dietary pattern, characterized by a high intake of chocolate-based products, breakfast cereals, water, and fruits, was not related to the risk of breast cancer in French-Canadian women, whereas Cui *et al* [15] found that a meat-sweet dietary pattern was associated with significantly increased breast cancer risk, though only among post-menopausal women (Q4 *vs*. Q1: OR = 1.3, 95% CI: 1.0–1.7). Differentially, the sweet dietary pattern established in this study was found to be protective against breast cancer. Overall, since the sweet foods involved in the previous mentioned dietary patterns was analyzed together with other foods (cereal, meat or vegetables), it is difficult to judge whether their observed effect was due to the intake of sweet food/beverages or that of other kinds of food eaten together [30]. According to the Chinese dietary culture, compositions in different kinds of foods could interact with each other and accordingly strengthen or weaken the health effect of foods [31]. Therefore, more studies are called to further testify the association between a sweet dietary pattern and breast cancer.

Although vegetables are generally believed to be beneficial to protect against certain cancers [32], there is still no consistency achieved in the relationship between dietary patterns in which more vegetables or fruit intake is involved and breast cancer. Some studies reported that a high consumption of vegetables had a protective effect against breast cancer [17, 24, 33]. Meanwhile, other researchers suggested that an increased consumption of vegetables and fruits was not associated with the risk of breast cancer [25, 34]. A prospective study performed in Japan [35] reported that there was no significant association between “vegetable pattern'' (mainly constitute of vegetables, potatoes, seaweed, tofu, fruits, *etc*.) and breast cancer, which is consistent with the outcomes that we observed in this analysis. Two studies on dietary patterns performed in other areas of China also found no association of high vegetables consumption and breast cancer [15, 18]. Sant *et al* [33] reported that only raw vegetables could protect against certain subtypes of breast cancer. Studies reporting a marked association between a vegetable dietary pattern and breast cancer risk were mostly performed in those countries, where vegetables usually are eaten raw. However, in the Chinese diet, vegetables are generally eaten after cooking, which may partially explain the null association that we observed.

Compared to Western countries, highly salted foods are commonly consumed in some Asian countries, such as Japan and China [27, 36]. Few studies have examined the association between a salty dietary pattern and breast cancer, and salted foods are hypothesized to increase the risk of some certain cancers as they may contain possible carcinogens, *i*.*e*., nitrates, which could be produced in the salting process. Nevertheless, we found no association between salty dietary pattern and breast cancer in this study. Maybe this is because that salt, rather than salted foods, is the main source of salt intake in Chinese diet [37].

A population-based study design, strictly adhering to a standard protocol for data collection, a relatively large sample size, together with other strict quality control measures (randomly matching cases and controls, training all researchers, regular evaluation on the quality of collected data, *etc*.), this study provides reliable evidence on the association of dietary patterns and breast cancer among Chinese women. Nonetheless, we should keep in mind that the dietary data collected through face-to-face questionnaire interview may not exactly reflect the real diet due to recall bias [38]. Even though all cases were newly diagnosed, it is possible that participants may change their dietary habits after being diagnosed with breast cancer. Secondly, specific breast cancer subtypes [*e*.*g*. estrogen receptor negative (ER-) and progesterone receptor positive (PR+)], which had been proven to be related to different dietary patterns [15, 26, 33], was not taken into consideration in this analysis due to the limitation of available data. Furthermore, we conducted this study in an area where people rely heavily on rice in their daily diet, while recognizing that China is characterized by remarkable cultural and dietary diversity. It is possible that the findings are not applicable to minority dietary cultures within China. Last but not least, available evidence on the possible mechanism of the observed associations of dietary patterns with breast cancer is still sparse and a potential protective effect of the traditional Chinese pattern needs to be further studied.

To conclude, the traditional Chinese dietary pattern (characterized by a relatively balanced intake of various nutrients) and a sweet pattern may protect against breast cancer among women in China, whereas a vegetarian pattern and a salty pattern appear not to be associated in this population.
